# Late-onset sepsis and mortality among neonates in a Brazilian Intensive Care Unit: a cohort study and survival analysis

**DOI:** 10.1017/S095026881900092X

**Published:** 2019-05-29

**Authors:** F. T. M. Freitas, A. F. O. L. Araujo, M. I. S. Melo, G. A. S. Romero

**Affiliations:** 1 Hospital Materno Infantil de Brasília, Health Secretariat of Distrito Federal, Brasilia, Brazil; 2 Nucleo de Medicina Tropical, University of Brasilia, Brasilia, Brazil

**Keywords:** Brazil, healthcare-associated infections, late-onset neonatal sepsis, survival analysis, very low birth weight infants

## Abstract

A cohort study was performed from January 2014 to December 2016 in a Brazilian neonatal intensive care unit, including neonates with high risk for infection and death. We estimated bloodstream infection (BSI) incidence and conducted a survival analysis, considering the time to death and to the first episode of BSI as outcomes, comparing very low birth weight (VLBW) neonates with the remaining neonates. An extended Cox model was performed and the hazard ratio (HR) was calculated for different time periods. The study had 1560 neonates included, the incidence and the incidence density of BSI was 22% and 18.6 per 1000 central venous catheter-days, respectively. Considering VLBW neonates as the reference group, the HR for time to death was 4.06 (95% CI 2.75–6.00, *P* < 0.01) from day 0 to 60 and for time to the first episode of BSI was 1.76 (95% CI 1.31–2.36, *P* < 0.01) from day 0 to 36. Having the heavier neonates group as reference, the HR for time to the first episode of BSI was 2.94 (95% CI 1.92–4.34, *P* < 0.01) from day 37 to 90. Late-onset neonatal sepsis prevention measures should consider the differences in risk during time, according to neonates' birth weight.

## Introduction

Brazil is a middle-income country that successfully reduced child mortality in the last decades, achieving the fourth millennium development goal in 2015. Nonetheless, it was based mainly on the reduction of post-neonatal mortality. As a result, neonatal mortality currently accounts for 69% of deaths in children below 1 year of age in the country [1]. Thus, in order to sustain infant mortality reduction, prevention strategies should focus on the neonatal period. The three main causes of death in the neonatal period in the country, according to the national mortality system (*Sistema de Informação sobre Mortalidade* – SIM), are prematurity, congenital anomalies and infection, the latter being the main cause of death in the late neonatal period (7–27 days of life) [1].

Neonatal sepsis is defined as a systemic infection occurring in the first 28 days of life. There are two patterns of disease: early-onset neonatal sepsis (EOS), variably defined as occurring within 48–72 h after birth and late-onset neonatal sepsis (LOS), occurring thereafter. Microorganisms responsible for EOS are of maternal origin, acquired hours or days before or during delivery from the birth canal. Neonates with EOS may have a history of prolonged rupture of membranes, preterm onset of labour, chorioamnionitis and peripartum maternal fever [2]. On the other hand, LOS is caused by microorganisms acquired in the community or during medical care in those who require hospitalisation. LOS is an important cause of morbidity and mortality in very low birth weight (VLBW) neonates and neonates with complex congenital anomalies during their stay in the neonatal intensive care unit (NICU) [3]. In Brazil, as 98% of births happen in the hospital [4], microorganisms acquired during the care given to hospitalised neonates are the main cause of LOS in this population. The incidence of LOS is inversely related to gestational age and birth weight [5]. Numerous risk factors are related to LOS, including prematurity and low birth weight, prolonged use of intravascular access, mechanical ventilation, total parenteral nutrition and exposure to broad spectrum antibiotics [5–8]. The incidence of LOS among VLBW neonates in a large number of NICUs in North America is 12% and in Germany is 15% [9, 10]. However, in Brazil, the incidence of LOS in VLBW population reaches 47%, being 24% confirmed by blood culture, according to data from the Brazilian Neonatal Research Network [11].

Therefore, in a post-2015 development agenda, recognising who are the neonates dying and becoming infected in the NICU and when these outcomes happen is important to establish strategies to reduce infant mortality in a middle-income country, such as Brazil. Thus, the objectives of this study were: (1) to estimate the incidence of LOS and its characteristics, distribution and the antimicrobial resistance profile of the microorganisms involved; (2) to estimate mortality rate according to birth weight; and (3) to compare the survival time from birth to death and from birth to the first bloodstream infection (BSI) episode among neonates admitted in a regional referral NICU, according to birth weight.

## Methods

### Design and location of study

This was a prospective cohort study, which included patients admitted from January 2014 to December 2016, at a public maternity hospital, located in Brasilia, Brazil. The hospital is a reference for care of preterm neonates and neonatal surgery, receiving high-risk pregnant women and patients from other services, covering an area of Brasilia and its surroundings, with approximately 4 million inhabitants [12]. The neonatology service has a capacity of 30 intensive care beds and 16 intermediate care beds. Intermediate care beds are for neonates who do not require mechanical ventilation or continuous positive airway pressure support and have no need for vasoactive or inotropes drugs to sustain blood pressure. There is a ratio of one doctor for every five beds, one nurse for every 10 beds in both intensive and intermediate care and one nursing technician for every two or three beds in intensive care and for every five beds in intermediate care. There are two hand hygiene sinks for every 10 beds and a 70% alcohol solution for hand hygiene is available for each bed. Hospital staff is encouraged to wash their hands according to the World Health Organization (WHO) five moments of hand hygiene [13]. The hospital infection control practices include active surveillance of healthcare-associated infections (HAI), antimicrobial stewardship program, management of infections and precautions, training of healthcare workers, among other good practices of infection control.

### Surveillance and definitions of HAI

Infections were actively detected through the result of laboratory cultures, antimicrobial prescription and during a multidisciplinary visit with the neonatology team. The infection control team performed HAI surveillance daily according to the Brazilian Agency of Sanitary Surveillance (*Agência Nacional de Vigilância Sanitária – ANVISA*) guidelines [14]. All neonates admitted to NICU who met one of the following criteria were the target of surveillance and, thus, included in the study: (1) birth weight <1500 g; (2) use of mechanical ventilation; (3) use of central venous catheter (CVC) (including umbilical catheter, peripherally inserted central catheter, CVC inserted by puncture or vein dissection); (4) underwent surgery; and (5) had any antimicrobial prescribed. These neonates were followed until they were discharged from the hospital or until they reached 90 days old, according to ANVISA guidelines.

The definitions of HAI were based on the ANVISA criteria [14]. Congenital infections (such as cytomegalovirus, toxoplasmosis, syphilis, rubella, among others) and infections of neonates admitted from home or from other hospitals that occur within the first 48 h of hospitalisation were not included in the HAI surveillance. HAI were classified into two groups: (1) early-onset, that manifest in the first 48 h of life caused by a microorganism of maternal origin, and (2) late-onset, that manifest after 48 h of life caused by a microorganism of hospital origin. We excluded early-onset HAI from this analysis and reported the rates of BSI, the main late-onset HAI during the neonatal period. The definitions of BSI are given in Table 1.

**Table 1.** Late-onset bloodstream infection criteria according to the Brazilian Agency of Sanitary Surveillance

Laboratory-confirmed BSI
Criteria 1: A positive blood culture from a non-skin contaminant microorganism and not related to an infection at another site. Criteria 2: Signs and symptoms not related to a non-infectious cause and to an infection at another site AND a common skin contaminant microorganism cultured from two or more blood cultures drawn on separate occasions OR coagulase-negative staphylococci cultured from a single blood culture if a central venous catheter is present.
Clinically-confirmed BSI
Signs and symptoms not related to a non-infectious cause and not related to an infection at another site AND a haematological score of Rodwell [15] ⩾3 or C-reactive protein (CRP) >10 mg/l AND a blood culture not done or no microorganisms detected in blood AND physician instituted treatment for sepsis.

Signs and symptoms: temperature instability, bradycardia, apnoea, poor feeding, respiratory distress, glucose intolerance, haemodynamic instability or diminished spontaneous activity/lethargy.

### Data collection and procedures

An infection control nurse collected maternal data: mother's age, gestational age, time of amniotic membranes rupture and maternal intercurrences such as chorioamnionitis; and neonatal data: date of birth, date of hospital admission, type of delivery, sex, birth weight, Apgar score, if there was any surgical pathology or congenital anomaly, use of any invasive medical device such as CVC and mechanical ventilation, antimicrobial use, culture results, date of discharge and outcome (hospital discharge or death). Gestational age was assessed primarily by the date of the last menstrual period. When this information was not reliable, gestational age was assessed by a first trimester gestational ultrasound when available, otherwise by postnatal examination, according to Capurro score or the new Ballard score if very preterm [16, 17].

Neonates with clinical suspicion of infection had their blood samples collected in the volume of 1 ml and immediately inoculated in bottles for blood culture. Samples were initially submitted to a continuous automated system for bacterial detection (Bactec fluorescent series system^®^ Becton Dickinson Microbiology System). Then, an automated bacterial identification panel (MicroScan Walk-Away^®^ Dade Behring Inc.) was used to identify the bacterial species and perform the antibiotic susceptibility. The laboratory used the Clinical and Laboratory Standards Institute (CLSI) manual for interpretation of the minimal inhibitory concentration [18].

### Statistical analysis

The total cumulative incidence of primary BSI was calculated using two different numerators: the total number of episodes of BSI or the number of patients with BSI (regardless of the number of episodes of BSI per patient), divided by the total number of patients admitted to the unit during the study period, multiplied by 100. The incidence density of BSI was calculated dividing the number of BSI by the total CVC-days, multiplied by 1000. HAI rates and descriptive results were stratified in two groups, according to birth weight, <1500 and ⩾1500 g. The results were compared, considering a statistically significant value of *P* < 0.05, using the *χ*^2^ test for dichotomous variables and the Kruskal–Wallis tests for continuous variables, presented as the median and interquartile range (IQR). The antimicrobials resistance profile of microorganism isolated from blood cultures were described.

A survival analysis was performed with two different outcomes: (1) the time from birth to death and (2) the time from birth to the onset of the first episode of BSI. The independent variable was birth weight, which was categorised into two distinct groups: VLBW neonates (<1500 g birth weight) and remaining neonates (⩾1500 g birth weight). When the outcomes of interest were not observed during the period of follow-up (until discharge or up to 90 days of life), these observations were censored. Death was considered a competing risk event when the outcome was the time until the first episode of BSI.

The cumulative probability of each outcome of interest, death and presenting at least one episode of BSI, was calculated using the Kaplan–Meier method [19]. The medians in days until death and until the first episode of BSI, as well as the probability of survival at day 7, 28 and 90 of follow-up were calculated for both groups. The Kaplan–Meier curves were compared using the log-rank statistical test [20]. Using the Cox proportional hazards regression model [21], the hazard ratio (HR) was calculated between the survival curve of VLBW neonates and that of the other neonates, adjusted by the following variables: sex, Apgar score at 5 min, presence of congenital anomalies and previous exposure to antibiotics for early-onset sepsis.

The basic premise of the model, the proportionality of HRs between the two groups was verified by the log–log survival curves and by the Harrel and Lee test, which uses Schoenfeld's residuals for the variables included in the models [22, 23]. In the Schoenfeld residue analysis, the proportionality premise of the HR was considered violated when the Harrel and Lee test was statistically significant (*P* < 0.05). When this assumption was violated, an extended Cox model was performed, allowing the hazards to vary over time. The HR was considered constant at two different times of follow-up and the HR was calculated for each period separately. Ninety-five per cent confidence intervals were calculated for HR measures, considering a statistically significant value of *P* < 0.05. The database was built using EpiInfo 7 software (CDC, Atlanta, GA, USA) and the analysis was performed on STATA software version 11 (StataCorp, College Station, TX, USA).

The institution's medical ethics committee approved the study (CAAE 23200113.0.0000.5553; Número de parecer 484.015).

## Results

### Maternal and neonatal data

From January 2014 to December 2016, 1506 neonates, who met the criteria for inclusion in HAI surveillance, were admitted to the neonatal unit and included in the study, a total of 34 906 patient-days. Of these, 943 (63%) had a birth weight of ⩾1500 g and 563 (37%) <1500 g. The median gestational age was 33 weeks (IQR 30–37 weeks) and the median birth weight was 1820 g (IQR 1240–2680 g). Table 2 presents the baseline characteristics of VLBW neonates and neonates with birth weight ⩾1500 g. Besides a lower gestational age, the duration of amniotic membrane rupture, CVC use and mechanical ventilation were significantly more prolonged among VLBW neonates, while the presence of congenital anomalies and surgical pathology was significantly more frequent among neonates with birth weight ⩾1500 g.

**Table 2.** Baseline characteristics of a cohort of neonates, according to birth weight, admitted to a Brazilian NICU from January 2014 to December 2016.

Characteristic	Birth weight		*P* value
	<1500 g (*n*.%) *N* = 563	⩾1500 g (*n*.%) *N* = 943	
Sex			
Male	296 (53)	509 (54)	0.59
Female	267 (47)	431 (45)	
Unknown	0	3 (1)	
Gestational age (weeks)			
<28	177 (31)	1 (1)	<0.01
28–31	271 (48)	88 (9)	
32–36	109 (20)	429 (45)	
⩾37	6 (1)	425 (45)	
Type of delivery			
Caesarean	347 (62)	575 (61)	0.80
Vaginal	216 (38)	368 (39)	
Time of amniotic membrane rupture (hours)			
At delivery	393 (70)	616 (65)	<0.01
<18	67 (12)	165 (18)	
18–72	32 (6)	74 (8)	
>72	71 (12)	88 (9)	
Apgar score at 5 min			
⩽5	29 (5)	39 (4)	0.36
Congenital anomalies	25 (4)	367 (39)	<0.01
Surgical pathology	33 (6)	276 (29)	<0.01
Time of central venous catheter (days)			
0	14 (2)	239 (25)	<0.01
1–7	140 (25)	281 (26)	
8–14	118 (21)	150 (16)	
15–21	105 (19)	97 (10)	
⩾22	186 (33)	176 (19)	
Time of mechanical ventilation (days)			
0	203 (36)	535 (57)	<0.01
1–7	174 (31)	250 (26)	
8–14	67 (12)	72 (8)	
15–21	18 (3)	25 (3)	
⩾22	101 (18)	61 (6)	

Congenital anomalies included the major congenital anomalies of heart, central nervous system, gastrointestinal tract and urinary tract. The main surgical pathologies were gastroschisis, omphalocele, oesophageal atresia, diaphragmatic hernia, necrotising enterocolitis and bowel obstruction, among others.

### Incidence of infection

There were 714 late-onset HAI, and 318 (45%) were laboratory-confirmed BSI, 122 (17%) conjunctivitis, 117 (16%) clinically-confirmed BSI, 58 (8%) surgical site infections, 24 (3%) meningitis, 23 (3%) pneumonia, 18 (3%) necrotising enterocolitis and 34 (5%) other infections.

Of a total of 435 episodes of BSI, 271 neonates had one episode of BSI, 52 had two episodes, 12 had three episodes and six had four episodes. The total of CVC-days was 23 391. The incidence of BSI was 29 episodes or 22 patients infected per 100 admissions. VLBW neonates had 243 BSI episodes and 189 were infected; an incidence of 43 episodes or 34 patients infected per 100 admissions. Among neonates born with birth weight ⩾1500 g, there were 192 episodes and 152 infected patients; an incidence of 20 episodes or 16 patients infected per 100 admissions (*P* < 0.01).

The overall incidence density of BSI was 18.6 per 1000 CVC-days. It was 21.4 per 1000 CVC-days among VLBW neonates and 15.9 per 1000 CVC-days among neonates with birth weight ⩾1500 g (*P* < 0.01, Table 3). The median age at the time of the first episode of BSI was 17 days (IQR 7–36 days). It was 13 days (IQR 7–26 days) among VLBW neonates and 28 days (IQR 10–52 days) among neonates with birth weight ⩾1500 g (*P* < 0.01).

**Table 3.** Frequency of bloodstream infection in a cohort of neonates, according to birth weight, admitted to a Brazilian NICU from January 2014 to December 2016

Birth weight	BSI laboratory confirmed	BSI clinically confirmed	BSI total	CVC-days	Incidence density per 1000 CVC-days
<1500 g	172	71	243	11 365	21.4
⩾1500 g	146	46	192	12 026	15.9
TOTAL	318	117	435	23 391	18.6

### Relative frequency of isolated pathogens

There were 318 episodes of laboratory-confirmed BSI in this study, a total of 209 (66%) were Gram-positive bacteria, 80 (25%) were Gram-negative bacteria and 29 (9%) were fungi. Table 4 presents the total numbers of isolated pathogens and their distribution according to birth weight. Three (14%) isolates of *S*. *aureus* were resistant to oxacillin and none of the *Enterococcus faecalis* isolates were resistant to ampicillin or vancomycin. Among Gram-negative bacteria, seven (9%) were resistant to amikacin, 27 (34%) were resistant to third- and fourth-generation cephalosporins and no acquired resistance to carbapenems was observed.

**Table 4.** Pathogens isolated from blood cultures in a cohort of neonates, according to birth weight, admitted to a Brazilian NICU from January 2014 to December 2016

Pathogen	<1500 g *n* (%)	⩾1500 g *n* (%)	Total *N* (%)
Gram-positive			
Coagulase-negative staphylococci	94 (55)	64 (43)	158 (50)
* Enterococcus faecalis*	19 (11)	12 (8)	31 (10)
* Staphylococcus aureus*	8 (5)	13 (9)	21 (7)
Gram-negative			
* Klebsiella* sp.	10 (6)	15 (10)	25 (8)
* Enterobacter* sp.	11 (6.5)	10 (7)	21 (7)
* Serratia marcescens*	6 (3.5)	7 (5)	13 (4)
* Escherichia coli*	1 (0.5)	4 (3)	5 (2)
* Citrobacter freundii*	1 (0.5)	2 (1)	3 (1)
* Pseudomonas aeruginosa*	3 (2)	0	3 (1)
* Acinetobacter baumannii*	3 (2)	0	3 (1)
Others	2 (1)	5 (3)	7 (2)
Fungi			
* Candida* sp.	2 (1)	4 (3)	6 (2)
* Candida albicans*	4 (2.5)	7 (5)	11 (3)
* Candida guillermondii*	2 (1)	3 (2)	5 (1)
* Candida lusitaniae*	2 (1)	1 (0.5)	3 (1)
* Candida tropicalis*	1 (0.5)	1 (0.5)	2 (0.5)
* Candida parapsilosis*	2 (1)	0	2 (0.5)
Total	170 (100)	148 (100)	318 (100)

Coagulase-negative staphylococci included *Staphylococcus epidermidis*, *S. capitis*, *S. hominis*, *S. haemolyticus*, among others. The other Gram-negative bacteria included two isolates of *Stenotrophomonas maltophilia* and *Ralstonia picketii*; one isolate of *Morganella morganii*, *Pantoea agglomerans* and *Serratia liquefaciens*.

### Mortality

There were 201 (13%) deaths, and of these, 54 (27%) occurred during antimicrobial treatment and without other apparent cause. Mortality was higher among VLBW neonates compared to the remaining neonates, 22% and 8%, respectively (*P* < 0.01). The median age at death was 8 days of life (IQR 3–21 days) among all neonates; 6 days (IQR 2–12 days) among VLBW neonates; and 10 days (IQR 3–44 days) among neonates with birth weight ⩾1500 g (*P* < 0.01).

### Survival analysis

**Fig. 1.**
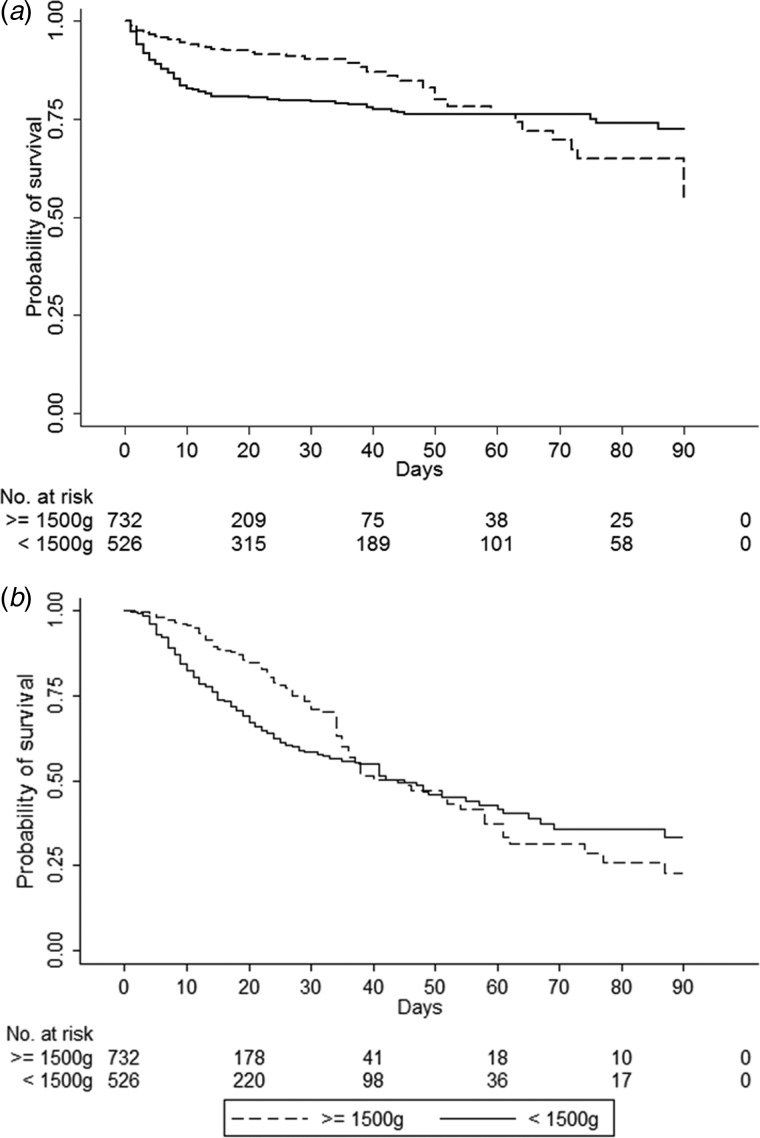
Kaplan–Meier estimates of survival in a cohort of high-risk neonates, according to birth weight, admitted to a Brazilian NICU from January 2014 to December 2016. (a) Time from birth to death as outcome. (b) Time from birth to the first episode of bloodstream infection as outcome.

For the survival analysis, we included 1237 neonates who were born in the hospital; admissions from transfers from other hospitals were excluded from this analysis. Figure 1 presents the survival curve by the Kaplan–Meier method for death and for the first episode of BSI as outcomes, between VLBW neonates and the remaining neonates. The times until death and until the first episode of BSI between the two groups were statistically different (log-rank test, *P* value < 0.01). In the analysis of the death as outcome, at the 7th and 28th day of life, the survival probabilities were 87% and 80% among VLBW neonates and 95% and 91% among the remaining neonates, respectively. However, it equalled at the 60th day of life, and at the end of the 90th day of follow-up, the probability of survival was 72% among VLBW neonates and 55% among the remaining neonates. In the analysis of the first episode of BSI as outcome, at the 7th and 28th day of life, the probabilities of survival without BSI were 89% and 59% among VLBW neonates and 97% and 75% among the remaining neonates, respectively. However, it equalled at the 36th day of life and at the end of the 90th day of follow-up, the probability of survival without BSI among VLBW neonates was 33% and 23% among the remaining neonates.

As the survival curves intersected, the premise of proportionality of the hazards between the two groups was violated (as it was ascertained by the analysis of log–log survival curves and Schoenfeld residues, data not shown). Therefore, an extended Cox model was performed to evaluate the HR in two different moments according to the outcome: for time from birth to death, the HR was calculated from 0 to 60 days and from 61 to 90 days; and for time from birth to the first episode of BSI, the HR was calculated from 0 to 36 days and from 37 to 90 days. The HR (adjusted for sex, Apgar score, presence of congenital anomaly and previous use of antibiotic for early-onset sepsis) estimated for time from birth to death among VLBW neonates compared to the remaining neonates during the first 60 days of life was 4.06 (95% CI 2.75–6.00; *P* < 0.01). Comparing heavier neonates with VLBW neonates during the days 61–90 yielded an HR of 2.22 (95% CI 0.56–8.33, *P* = 0.26). The estimated adjusted HR of the time from birth to the first episode of BSI (considering death as a competing risk) comparing VLBW neonates with the remaining neonates during the first 36 days of life was 1.76 (95% CI 1.31–2.36; *P* < 0.01). Comparing heavier neonates with VLBW neonates during the days 37–90 yielded an HR of 2.94 (95% CI 1.92–4.34; *P* < 0.01).

## Discussion

Our study demonstrates a high burden of LOS among neonates admitted to a NICU in Brazil, especially among those diagnosed with VLBW. Regarding the onset of infection, 22% of neonates presented at least one episode of BSI, but the incidence was higher (34%) when considering only VLBW neonates, with a median occurrence in the 13th day of life, being a clear threat to those neonates who survived the complications of preterm birth in the first 2 weeks of life. These figures are lower than those reported by the Brazilian Neonatal Research Network, which found an incidence of 47% of BSI among VLBW neonates [11]. The incidence density of BSI was in the same range of that found in other Brazilian studies, which varied from 13.3 to 25.3 BSI per 1000 CVC-days [24–27], and similar to those reported by the International Nosocomial Infection Control Consortium (INICC), which involved 48 NICU from middle- and low-income countries (18 from Latin America), that found a pooled mean of 16.37 BSI per 1000 CVC-days [28]. However, these numbers are higher than that observed in developed countries. For instance, data from national surveillance systems of HAI show a BSI incidence density of 1.3 per 1000 CVC-days among all neonates in the USA [29], and 8.6 per 1000 CVC-days among VLBW neonates in Germany [30]. Crude mortality in this cohort was 13%; it is difficult to determine the role of LOS as the primary cause of death in this population due to the complications of prematurity and congenital anomalies. Nonetheless, we estimated a fatality rate of 27% for BSI among those infected neonates, considering death during antimicrobial treatment and with no other acute complication for death.

We hypothesised that death and the development of BSI affected neonates differently according to birth weight during their stay in the NICU. Therefore, we performed a survival analysis that found that mortality among VLBW neonates was much higher than that observed in other neonates (22% *vs.* 8%) and affected this population earlier (median at the sixth day of life). In fact, among VLBW neonates, the hazard for the time to death was four times higher compared to heavier neonates during the first 60 days of life, with no difference thereafter. This fact highlights the urgent need to prevent preterm birth as a strategy to reduce neonatal mortality. This is especially important in Brazil, where preterm birth has increased over the last years [31], reaching 11.9% of all births [1], twice of what is observed in developed countries (5.5%) [32]. Improving antenatal care, with special attention to poverty-related maternal conditions, such as infections during pregnancy, pre-eclampsia, gestational diabetes, vaginal bleeding, low body mass index, smoking and alcohol or drug abuse would reduce preterm birth. Moreover, reduction in caesarean delivery would also contribute to reduce preterm birth in Brazil, where the rate of caesarean delivery is one of the highest in the world (55%) [33].

On the other hand, the dynamics of LOS was somewhat different; it affected VLBW neonates earlier compared to the other neonates and the HR for the time to the first episode of BSI in this group was 1.76 in the first 36 days of life. Nonetheless, that effect reverses from the 37th day of life until the end of follow-up at the 90th day of life, and the HR for the time to the first episode of late BSI became even higher, 2.94, comparing heavier neonates, as the reference group, to VLBW neonates. Since VLBW neonates are exposed to healthcare-associated procedures, such as deep vascular access and mechanical ventilation as early as in the first week of life, which is their most delicate moment in the NICU, they have a higher risk of developing infection in the first month of life. On the other hand, heavier neonates have a higher proportion of congenital anomalies and surgical pathologies, requiring deep vascular access and mechanical ventilation for the management of complex pathologies and surgical complications at later times of their hospitalisation in the NICU, presenting HAI after the first month of life. These data demonstrate the complexity of LOS. Its early identification can be difficult because of its unspecific signs and symptoms and delay in culture results. Attempts to improve diagnostics sensitivity with sepsis scores and algorithms incorporating different combinations of inflammatory response parameters, laboratory assessments and physical examination findings have been developed, but a single score has not proven to be consistently reliable. In a European prospective study that enrolled 113 neonates, the positive predictive value of the criteria developed by an expert panel to identify culture-proven late-onset sepsis was 61% (95% CI 52–70) and 43 different empirical antibiotics regimens were noted [34]. Thus, LOS prevention is paramount; there is enough evidence to suggest that the implementation of care bundles reduce central line-associated BSI in neonatal units [35].

Our results are important because they can refine HAI prevention strategies according to birth weight, since the risk of infection varies differently during NICU stay in these two groups. Additionally, they cover the unfinished agenda of reducing neonatal deaths and disabilities worldwide, especially in countries that expanded access to health care and reduced post-neonatal mortality, such as Brazil. The WHO published ‘Every Newborn: an action plan to end preventable deaths’, to reach the target of 10 or less neonatal deaths per 1000 live births by 2035 [36] and the United Nations have developed Sustained Development Goals, including goal number three, which aim to reduce neonatal mortality to at least as low as 12 per 1000 live births by 2030 [37]. This will be accomplished by investing in quality care around the time of birth and special care for sick and small neonates. Improvement of quality and equity of care provided for sick and small neonates affects health outcomes in lower-mortality settings, and its implementation represents a new challenge for developing countries [38]. Future research should evaluate the effectiveness of quality improvement methods aimed at HAI prevention, including hand hygiene compliance, bundles of BSI prevention, safe surgery and antimicrobial stewardship programmes applied to scenarios similar to observed in our study. Therefore, we need investments to overcome constraints in staff personnel; inadequate nurse-to-patient ratio is frequent and it has proved to be an isolated risk factor for HAI [39]; besides the availability of resources and supplies to perform high-quality neonatal care.

Our study had limitations, although the great majority of births in Brazil occur in the hospital, we restricted our population of study to neonates who had access to intensive care medicine, risking losing cases that died before reaching the NICU. Additionally, we lost follow-up of neonates transferred to other hospitals that could have experienced one of our outcomes: death or an episode of BSI. However, neonates were only transferred to other hospitals to continue their treatment when they had already improved clinically. We did not have a protocol for blood culture sampling, which may explain the high proportion of clinically confirmed BSI and CoNS isolates that may have overestimated the number of infections. We rarely had two or more blood samples for culture, making it difficult to establish when a CoNS was a cause of infection or a sample contaminant [40]. Furthermore, we cannot generalise our results to different NICU, especially in such a large and diverse country as Brazil. Nevertheless, this was a prospective study carried out in a regional referral unit that concentrates preterm neonates and neonates with congenital anomalies and surgical pathologies, a population prone to infection and death, ideal for our study.

In conclusion, the rates of BSI reported in the NICU are high when compared to that observed in developed countries, and pose a challenge for middle-income countries that successfully reduced post-neonatal mortality, such as Brazil. If they want to keep with the reduction in infant mortality rates, they must invest in the quality of care and HAI prevention strategies must be adapted and evaluated, taking into account the different moments of risk that exist during hospitalisation between different groups of neonates according to birth weight.
